# Reduction of Preneoplastic Lesions Induced by 1,2-Dimethylhydrazine in Rat Colon by Maslinic Acid, a Pentacyclic Triterpene from *Olea europaea* L.

**DOI:** 10.3390/molecules24071266

**Published:** 2019-04-01

**Authors:** M. Emília Juan, Glòria Lozano-Mena, Marta Sánchez-González, Joana M. Planas

**Affiliations:** 1 Departament de Bioquímica i Fisiologia, Universitat de Barcelona (UB), Av. Joan XXIII 27-31, 08028 Barcelona, Spain; gloria.lozano.mena@gmail.com (G.L.-M.); chan_marta@hotmail.com (M.S.-G.); 2 Institut de Recerca en Nutrició i Seguretat Alimentària (INSA), Universitat de Barcelona (UB), Av. Prat de la Riva 171, 08921 Santa Coloma de Gramanet, Spain

**Keywords:** aberrant crypt foci, colon cancer, mucin depleted foci, maslinic acid, dimethylhydrazine

## Abstract

Maslinic acid triggers compelling antiproliferative and pro-apoptotic effects in different human cancer cell lines. Hence, the chemopreventive activity was investigated on early stages of carcinogenesis induced by 1,2-dimethylhydrazine (DMH) which is a model that mimics human sporadic colorectal cancer. Male Sprague-Dawley rats were orally administered either maslinic acid at 5, 10 or 25 mg/kg dissolved in (2-hydroxypropyl)-β-cyclodextrin 20% (*w*/*v*) or the solvent for 49 days. After one week of treatment, animals received three weekly intraperitoneal injections of DMH at the dose of 20 mg/kg. Maslinic acid reduced the preneoplastic biomarkers, aberrant crypt foci (ACF) and mucin-depleted foci (MDF), already at 5 mg/kg in a 15% and 27%, respectively. The decline was significant at 25 mg/kg with decreases of 33% and 51%, respectively. Correlation analysis showed a significant association between the concentrations of maslinic acid found in the colon and the reduction of ACF (r = 0.999, *p* = 0.019) and MDF (r = 0.997, *p* = 0.049). The present findings demonstrate that maslinic acid induced an inhibition of the initiation stages of carcinogenesis. The assessment of this pentacyclic triterpene at the colon sheds light for designing diets with foods rich in maslinic acid to exert a chemopreventive activity in colorectal cancer.

## 1. Introduction

**Figure 1:**
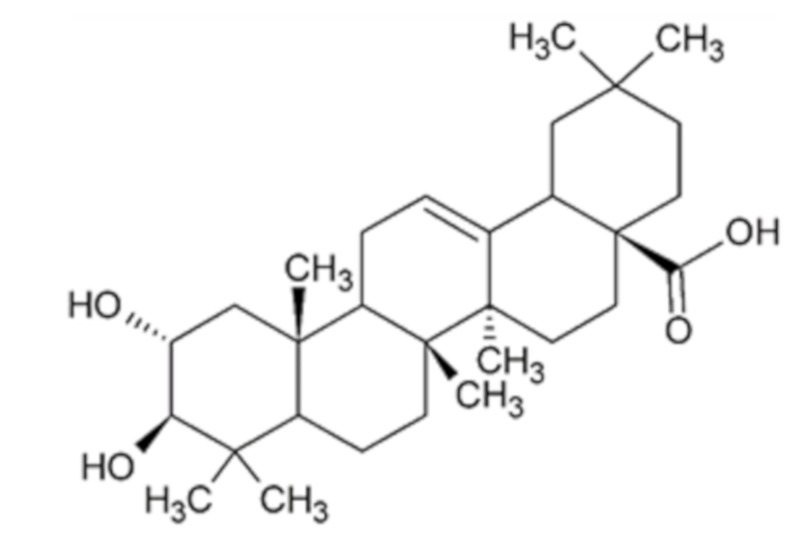
Chemical structure of maslinic acid.

Colorectal cancer (CRC) is one of the most frequent malignancies in the world, ranking third in incidence and second in mortality in both genders [1]. Epidemiological studies support that lifestyle along with nutritional interventions can prevent CRC and that Mediterranean diet is associated with a lower prevalence of this disease in humans of all races [2,3,4]. This eating pattern is characterized by a high consumption of fruits, vegetables and legumes that provides a plethora of bioactive compounds with antitumoral activity [2,3,4]. Among them, stand out resveratrol contained in grapes [5], sulforaphane in cruciferous vegetables [6] or luteolin in celery and parsley [7]. Hence, chemoprevention with natural compounds is currently proposed as a dietary strategy for the control and constraint of carcinogenesis [8]. Therefore, here we focus our attention on maslinic acid, also known as crategolic acid or 2α,3β-dihydroxyolean-12-en-28-oic acid (Figure 1) with molecular weight of 472.7 g/mol. This phytochemical belongs to the group of pentacyclic triterpenes and is broadly distributed in nature, being found in different foods such as olives, spinaches, eggplants, chickpeas, lentils, kiwi and pomegranates as well as in plants used in traditional Asian medicine for the treatment of different affections [9]. In this sense, the leaves of banaba or *Lagerstroemia speciosa* L. have been used for the treatment of diabetes, *Crataegus monogyna* L. commonly known as hawthorn, constitute a remedy for cardiovascular diseases, and the leaves of loquat or *Eriobotrya japonica* L. have been employed as antitussive and anti-inflammatory for chronic bronchitis [9]. 

Maslinic acid is gaining interest due to its lack of harmful effects [10] together with its multiple beneficial activity on health, essentially antidiabetic, antioxidant, anti-inflammatory, cardioprotective, neuroprotective and antitumoral [9]. Among them, it its chemopreventive activity in the human colon adenocarcinoma cell lines HT29 and Caco-2 cells [11,12,13] is worth mentioning. Moreover, it was also demonstrated that after the oral administration of maslinic acid, high concentrations were found in the colon due to its low bioavailability [14]. Taking together these results, they suggest that the distal part of the intestine could be a target organ where maslinic acid can exert its beneficial effects. 

Although the promising activities demonstrated in vitro, the antitumoral action in colon cancer in vivo has only been evaluated in male *Apc*^Min/+^ mice which is a genetic model that mimics the familial cancers such as human familial adenomatous polyposis (FAP) and hereditary non-polyposis colon cancer. Results indicated that maslinic acid at 100 mg/kg of diet suppressed polyp formation by a 54% [15]. However, the most common cancer is the non-familial colorectal type, which occurs sporadically and can be induced in animal models with the use of carcinogens [16]. Therefore, the chemopreventive effect of maslinic acid was investigated on colonic preneoplastic lesions induced by the administration of 1,2-dimethylhydrazine (DMH) in rats. To that end, maslinic acid was administered at 5, 10 and 25 mg/kg, being the lower dose easily attained following a Mediterranean dietary pattern. Maslinic acid efficacy was assessed in terms of appearance of aberrant crypt foci (ACF) that were determined as biomarker of cancer risk and mucin depleted foci (MDF) measured as biomarker of dysplasia since they harbor characteristics comparable to microadenomas [17]. The reduction of these preneoplastic biomarkers by the phytochemical will confirm its action in vivo. In addition, the relationship between the colonic concentrations of maslinic acid and the occurrence of ACF and MDF were assessed. For that purpose, maslinic acid was quantified in the colon content by LC-APCI-MS analysis and established the correlation between its concentrations in the colon and the reduction of preneoplastic biomarkers. The results provide new insights not only on the maslinic effects on gut health but also a thorough basis to allow a dietary recommendation of the intake of foods with a high content of this pentacyclic triterpene.

## 2. Results

### 2.1. Body Weight, Food and Water Consumption, and Food Conversion Efficiency

Careful observation of the animals during the experimental period showed no mortality or adverse effects. The consistency of stools was pelleted and firm, with no visible differences throughout the seven groups.

**Figure 2:**
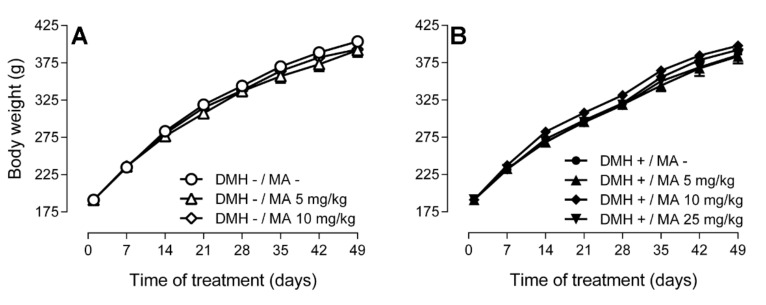
Body weight of Sprague-Dawley rats (**A**) that did not receive the carcinogen and were orally administered with solvent or maslinic acid at 5 or 10 mg/kg, whereas (**B**) displays the groups that received 1,2-dimethylhydrazine (DMH) once a week for three weeks long with solvent or maslinic acid at 5, 10 or 25 mg/kg. Results are expressed as mean ± standard error of the mean (SEM) (*n* = 6–8) and were analyzed by two-way ANOVA, followed by Tukey’s multiple comparisons test. No significant differences (*p* > 0.05) were found between groups submitted at the different treatments.

Body weight was not affected by the three consecutive intraperitoneal injections of DMH compared to the controls. Moreover, the daily oral administration of maslinic acid at the doses of 5, 10 or 25 mg/kg for 49 days did not modify body weight with respect to the control groups (Figure 2).

**Figure 3:**
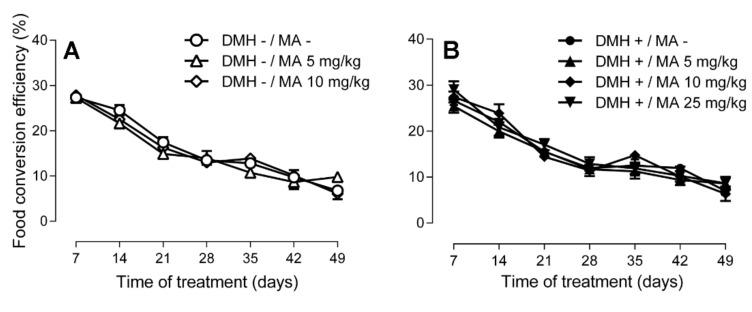
Food conversion efficiency of Sprague-Dawley rats (**A**) that were not intraperitoneally injected with the carcinogen and were orally administered with solvent or maslinic acid at 5 or 10 mg/kg, whereas (**B**) depicts the groups that received DMH once a week for three weeks along with solvent or maslinic acid at 5, 10 or 25 mg/kg. Results are expressed as mean ± SEM (*n* = 6–8) and were analyzed by two-way ANOVA, followed by Tukey’s multiple comparisons test. No significant differences (*p* > 0.05) were found between groups at the different treatments. Differences over time: DMH−/MA−, DMH−/MA 10, DMH+/MA10, 7 d = 14 d > 21 d = 28 d = 35 d = 42 d = 49 d; DMH+/MA−; DMH−/MA 5, DMH+/MA5, DMH+/MA25, 7 d > 14 d > 21 d = 28 d = 35 d = 42 d = 49 d.

The seven groups did not differ in food and water consumption (data not shown). Figure 3 displays the food conversion efficiency (FCE) that showed the same pattern in all experimental groups. FCE was highest during the first week, decreased during the second and third week, and remained constant thereafter, with no significant differences between groups.

### 2.2. Aberrant Crypt Foci

**Figure 4:**
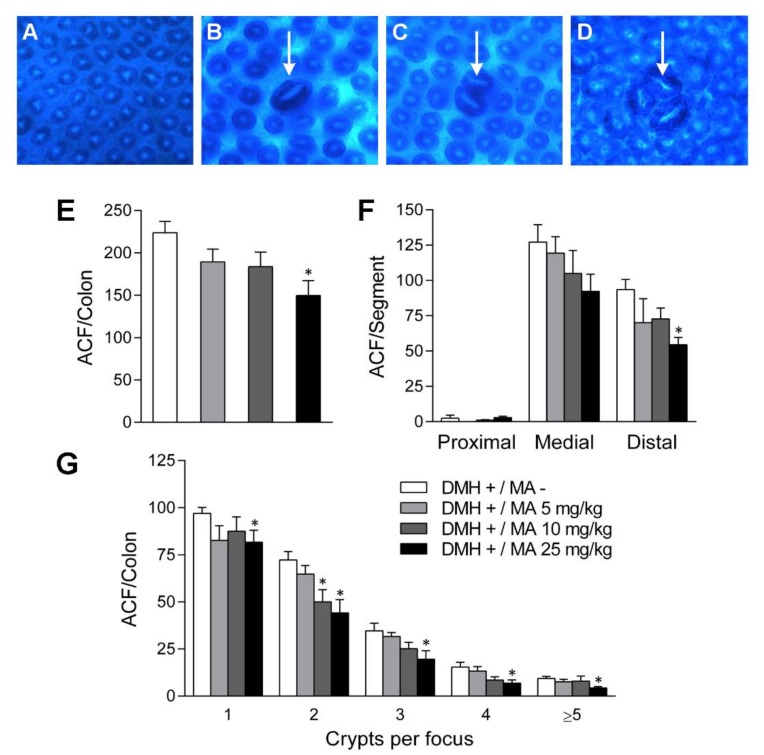
Aberrant crypt foci (ACF) observed under a light microscope after staining of the colon with methylene blue (magnification of ×10). The images show the whole mount colon of animals in (**A**) the negative control (DMH−/MA−) and the positive control (DMH−/MA+) showing a topographic view of ACF indicated by a white arrow, with one (**B**), two (**C**) or three crypts (**D**). The effects of maslinic acid at the doses of 0, 5, 10 and 25 mg/kg on the number of ACF in (**E**) total colon; (**F**) colonic segments and (**G**) number of crypt per focus in total colon. Results are expressed as mean ± SEM and were analyzed by the non-parametric Kruskal–Wallis test, followed by Dunn’s multiple comparisons test, *n* = 6–8. Asterisks indicate different from the control group: * *p* < 0.05.

The colon mucosa of rats in the three DMH—groups, namely, intraperitoneally injected with EDTA 1 mmol/L (pH 6.5) and orally administered with either (2-hydroxypropyl)-β-cyclodextrin 20% (*w*/*v*) or 5 and 10 mg/kg of maslinic acid did not show any microscopically observable alterations compatible with the presence of ACF. Given that no preneoplastic lesions were found in the three groups that were injected with saline solution (Figure 4A), all subsequent analysis were restricted to the four groups that received DMH and developed ACF (Figure 4B–D).

The number of preneoplastic lesions in colon was higher in the DMH+/MA− group (224 ± 37 ACF), than in the maslinic acid treated animals. This pentacyclic triterpene reduced the lesions to 189 ± 40 (*p* > 0.05), 184 ± 38 (*p* > 0.05) and 150 ± 43 (*p* < 0.05), at the doses of 5, 10 and 25 mg/kg, respectively (Figure 4E). ACF followed a regional distribution along the colon that was similar in all groups. In the positive control, ACF were practically absent in the proximal segment, increased to 127.1 ± 12.4 in the medial segment and were 98.4 ± 6.32 in the distal part (Figure 4F). Maslinic acid diminished the formation of ACF at the three doses evaluated, being 25 mg/kg the most effective one with a count of 92.3 ± 12.1 in the medial (*p* > 0.05) and 54.5 ± 5.1 in the distal segments (*p* < 0.05). Noteworthy, at the three doses evaluated maslinic acid was more active in inhibiting ACF in the distal parts of the colon, with reductions of 25% (*p* > 0.05), 22% (*p* > 0.05) and 42% (*p* < 0.05) at 5, 10 and 25 mg/kg, respectively.

The number of AC in each focus or crypt multiplicity was also determined (Figure 4G). In the DMH+/MA− group, most of the lesions were formed by a single crypt (42%), followed by the number of foci containing 2 crypts (32%), while thereafter, the foci comprising 3, 4 and ≥5 crypts were progressively lower, appearing at a 15%, 7% and 4%, respectively. A similar distribution was observed in the treated groups, in which maslinic acid reduced the number of lesions (Figure 4G). The effect exerted in the foci with higher number of crypts, since those with 2 crypts decreased in a 31% (*p* < 0.05) and 39% (*p* < 0.05) at the doses of 10 and 25 mg/kg, respectively. This pattern was also repeated at the foci with 3 crypts that dropped in a 27% (*p* > 0.05) and 43% (*p* < 0.05) at 10 and 25 mg/kg, respectively. Remarkable is that maslinic acid also induced a significant decrease in the foci with 4 and ≥5 crypts at the dose of 25 mg/kg since these lesions were reduced in a 56% (*p* < 0.05) and 54%, respectively (*p* < 0.05).

Aberrant crypts (AC) in the colon of the animals that were injected intraperitoneally with DMH but did not receive maslinic acid were 445 ± 36. The oral administration of this compound reduced AC to 388 ± 31 (*p* > 0.05), 341 ± 42 (*p* > 0.05) and 258 ± 42 (*p* < 0.01), at the doses of 5, 10 and 25 mg/kg, respectively.

### 2.3. Mucin-Depleted Foci

**Figure 5:**
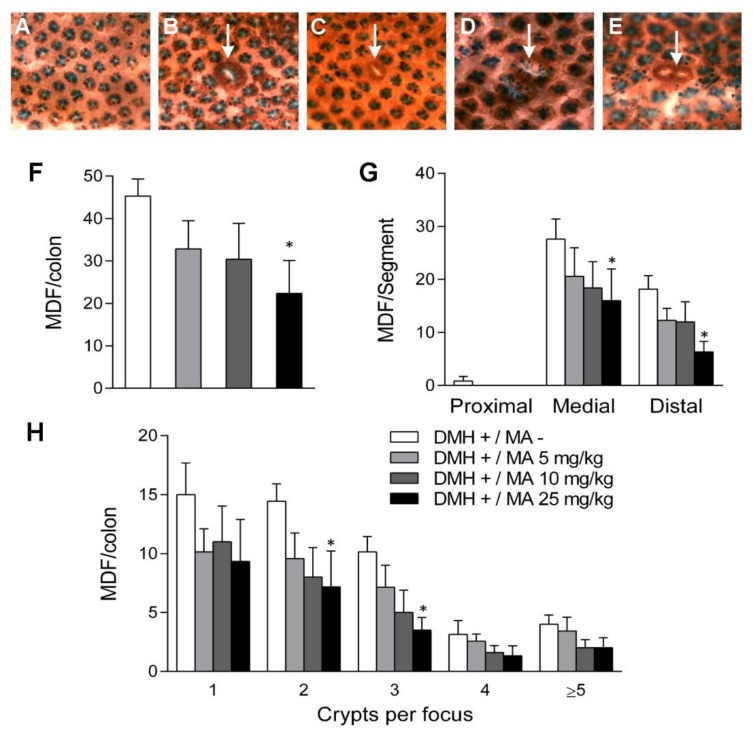
Mucin depleted foci (MDF) assessed after dying the colon with high-iron diamine/alcian blue/neutral red staining (HID-AB) and analyzed under a light microscope (magnification of ×10). Topographic features of the colonic epithelium of animals in (**A**) the negative control (DMH−/MA−) and the positive control (DMH+/MA−) displaying ACF with one (**B**) or two (**D**) crypts and MDF with one (**C**) or two crypts (**E**). The effects of maslinic acid at the doses of 0, 5, 10 and 25 mg/kg on the number of MDF in (**F**) total colon; (**G**) colonic segments and (**H**) number of crypt devoid of mucin per focus in total colon. Results are expressed as mean ± SEM and were analyzed by the non-parametric Kruskal–Wallis test, followed by Dunn’s multiple comparisons test, *n* = 6–8. Asterisks indicate different from the control group: * *p* < 0.05.

The oral administration of maslinic acid prevented the formation of dysplastic lesions or MDF which are aberrant crypts characterized by the loss of mucin production (Figure 5C,E). While in the DMH+/MA− group the number of MDF was 45.3 ± 4.0, the incidence decreased to 32.9 ± 6.6 (*p* > 0.05), 30.4 ± 8.5 (*p* > 0.05) and 22.3 ± 7.8 (*p* < 0.05), in the groups treated with 5, 10 and 25 mg/kg, respectively (Figure 5F). The depletion of mucins in aberrant crypts followed the same regional distribution observed for ACF (Figure 5G). Maslinic acid groups had fewer MDF than the DMH group, with the highest efficiency at the dose of 25 mg/kg.

The incidence of crypt multiplicity for 1, 2 and 3 crypts per focus was markedly lower in the maslinic acid groups than in the DMH+/MA− group (Figure 5H). Total mucin depleted crypts in the DMH+/MA− group were 105.4 ± 7.3, that were reduced to 78.1 ± 16.7 (*p* > 0.05), 61.2 ± 17.0 (*p* > 0.05) and 49.5 ± 18.1 (*p* < 0.05), in the groups that were orally administered with the doses of 5, 10 and 25 mg/kg of maslinic acid. Therefore, this bioactive compound was able to decrease the number of dysplastic mucin depleted aberrant crypts (MDAC) in colon by 26%, 42% and 53%, at the doses of 5, 10 and 25 mg/kg of maslinic acid.

### 2.4. Determination of AST and ALT

The hepatic enzymes were determined in order to assess the safety of experimental animals. Therefore, AST and ALT were analyzed in the three representative groups: the control rats, that did not receive neither the treatment, nor maslinic acid (DMH−/MA−), the animals challenged with the carcinogen that only received the solvent (DMH+/MA−), and the ones administered with the highest dose of maslinic acid at 25 mg/kg (DMH+/MA 25). The results obtained for AST and ALT indicate that the hepatic integrity of the animals is maintained throughout the experiment, since no significant differences were observed between groups (Table 1). 

### 2.5. Quantification of Maslinic Acid in Colon Content

**Figure 6:**
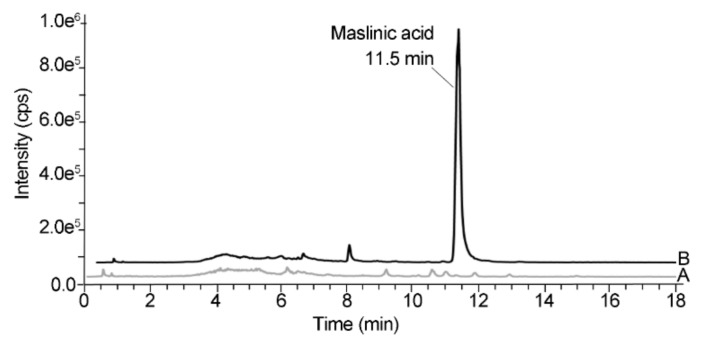
Representative liquid chromatography-mass spectrometry (LC-MS) chromatograms obtained in single ion monitoring (SIM) acquisition mode at *m*/*z* 471.3 of the colon content of (A) a rat from the DMH+/MA− group and (B) an animal from the DMH+/MA 10 mg/kg group.

Figure 6 depicts the typical chromatograms of the colon content from rats challenged with DMH that received the solvent or maslinic acid at the dose of 10 mg/kg for 49 days obtained between 18 and 20 h after the last oral administration.

The colon content of the rats in the DMH+/MA− group did not show any maslinic acid, whereas the animals orally administered with the pentacyclic triterpene showed a peak at 11.5 min that eluted free from other interfering peaks, thus indicating the selectivity of the analytical method. 

**Figure 7:**
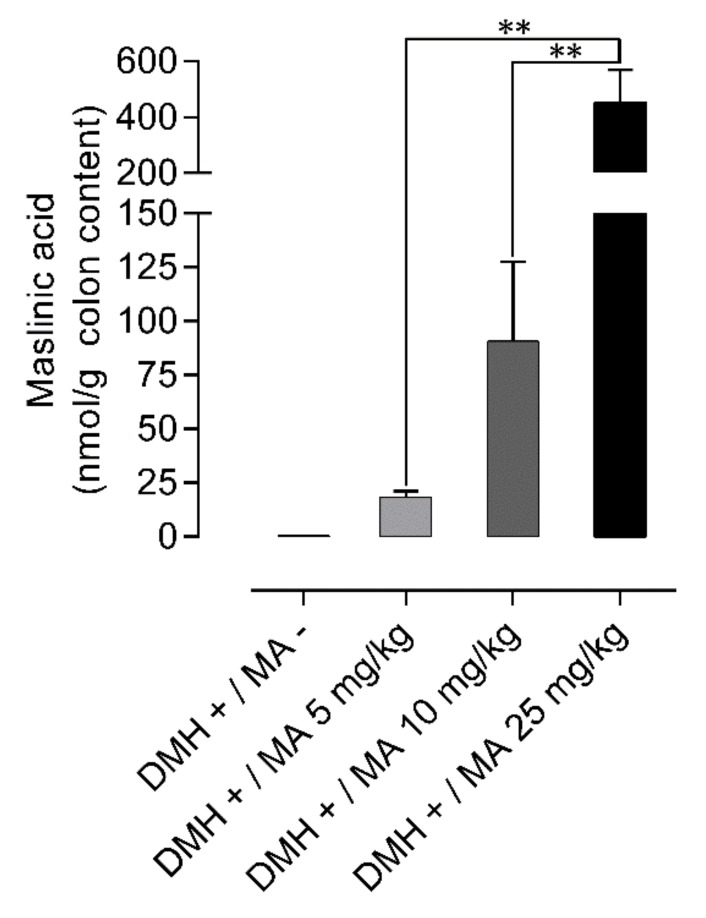
Concentrations of maslinic acid in the colon content of DMH-injected rats obtained 24 h after the last oral administration of the pentacyclic triterpene at the doses of 0, 5, 10 and 25 mg/kg after 49 days of treatment. Results are expressed as mean + SEM and were evaluated with one-way ANOVA and Bonferroni’s multiple comparisons test, *n* = 6–8. Asterisks indicate differences between doses: ** *p* < 0.01.

Maslinic acid was found in the colon content achieving concentrations of 18.4 ± 2.7 nmol/g; 90.7 ± 36.9 nmol/g and 453.6 ± 114.3 nmol/g in animals that received 5, 10 and 25 mg/kg (*n* = 6), respectively. The DMH+/MA− group was also analyzed, and no maslinic acid was found in any sample (Figure 7). 

The possible effect of DMH treatment on the concentrations of the pentacyclic triterpene that reached the colon was evaluated. Therefore, the content of the rats in the group that was not injected with the carcinogen but was orally administered throughout the experimental period with the pentacyclic triterpene at the doses of 10 mg/kg was analyzed. The concentration of maslinic acid in the colon was 86.4 ± 7.6 nmol/g (*n* = 6), which did not differ from the 90.7 ± 36.9 nmol/g achieved in the DMH+/MA 10 mg/kg group (*p* > 0.05). Given that DMH treatment did not modify the amounts of maslinic acid reaching the colon, the animals in the DMH−/MA 5 mg/kg were not included in the analysis.

### 2.6. Correlation between the Reductions of Preneoplastic Markers and Concentrations in the Colonic Content

**Figure 8:**
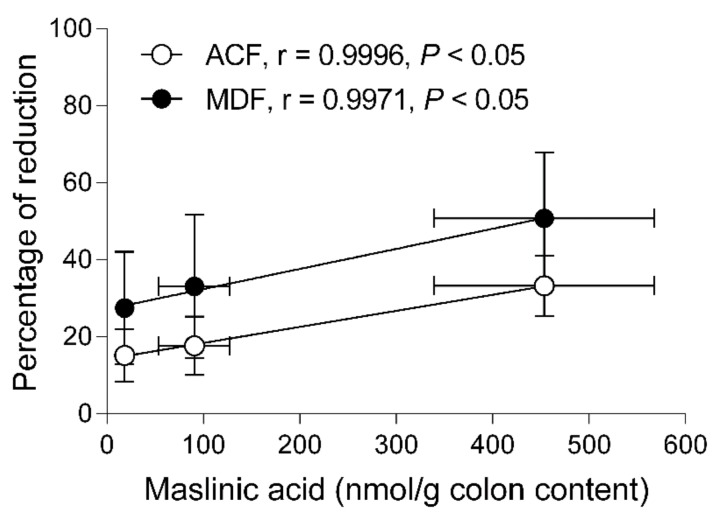
Pearson correlations of the reductions of aberrant crypt foci (ACF) and mucin depleted foci (MDF) versus concentrations of maslinic acid in the colonic content. Values are represented as means ± SEM. Horizontal bars are the SEM of the concentrations of maslinic acid in the colon (*n* = 6 for each dose). Vertical bars represent the SEM of the percentage of reduction of ACF (*n* = 6 for each dose) or MDF (*n* = 6 for each dose).

The concentrations of maslinic acid found in the colon content were determined to establish a correlation between the biological effects observed in the colon and the amounts of the bioactive compound in the site of action (Figure 8). The percentage of reduction of both, ACF and MDF versus the amount of maslinic acid in the colon content showed a strong uphill linear relationship with Pearson’s correlation coefficients higher than 0.99. The activity of maslinic acid in reducing preneoplastic lesions was parallel in the three doses studied but the effect of this pentacyclic triterpene was approximately 1.8-folds higher on reducing MDF than ACF.

## 3. Discussion

The effect of maslinic acid was evaluated in rats in a short-term assay in which precancerous lesions were induced by DMH. This animal model is one of the most frequently used for the study of chemopreventive agents since develops morphological and histological features similar to those observed in CRC, which is sporadic and the most common in humans [18]. DMH is an alkylating agent that produce free radicals that bind to DNA and cause mutations [16], specifically in the large intestine upon subcutaneous or intraperitoneal administration in a dose dependent manner [16,18]. The mechanisms postulated to cause these preneoplastic lesions were the accumulation of mutations that drive tumor initiation and then progression [19]. The carcinogen DMH cause mutations in the DNA affecting different genes. Between them, *K*-*ras* mutations were found to be frequent (between 20–40%) in the first step of CRC process, where ACF are hyperplastic. The mutation of this oncogene encode an intracellular signaling molecule that activate Ras and its down-stream signaling pathways, such as the Raf/MEK/MAPK and PI3K/Akt/PKB [17,20]. In addition, mutations of the β-*catenin* gene have been described in dysplastic MDF, being the activation of Wnt signaling by accumulation of β-*catenin* a major mechanism in the DMH-induced colon carcinogenesis model that leads to cell proliferation [21]. Mutations of *K-ras* and β-*catenin* may be involved in the up-regulation of NF-κB, cyclin D1, COX-2 and iNOS causing an increase in cell proliferation and a decrease of apoptosis [22].

The experimental design used in the present study consisted on three subcutaneous injections of DMH followed by an observation period of 4 weeks, resulted in the formation of preneoplastic lesions, including aberrant crypt foci (ACF) and mucin-depleted foci (MDF) [5]. ACF not only appears in murine animal models induced with carcinogens but also have been described in humans suffering from CRC and FAP [23]. ACF are the first lesions in the development of CRC and show a marked hyperplasia. MDF constitute a more advanced stage than ACF and are characterized by a scarce or absent production of mucins secreted by goblet cells, and exhibit dysplasia [18]. These crypts devoid of mucins have been identified in the colon of humans at high risk of cancer and are considered a hallmark of malignant potential [17]. ACF and MDF are considered as biomarkers of colon carcinogenesis and are used to evaluate the chemopreventive potential of bioactive compounds [24].

Interestingly, the administration of the carcinogen produced preneoplastic lesions in our murine model without any signs of toxicity confirming previous results [5]. When the chemopreventive agent, maslinic acid, was also tested, no adverse effects were observed. Body weight, food conversion efficiency and hepatic enzymes were affected neither by treatment with DMH nor by maslinic acid. These results are consistent with the lack of toxicity reported for this pentacyclic triterpene in different animal species [10,25,26] and humans [27].

In our study, the number of ACF and MDF developed in the positive control rats (DMH+/MA−) was in agreement with the results obtained previously in our group [5] as well as by other authors [18,24,28]. Our findings were also consistent in their distribution along the colon, mostly in the middle and distal segments [18,24,28]. Once the model was established, it was applied to the evaluation of the chemopreventive activity of maslinic acid. Our results indicate that this pentacyclic triterpene exerted a protective activity since ACF in total colon were reduced in a 15%, 18% and 33% at the doses of 5, 10 and 25 mg/kg, respectively. Multiplicity was also affected by maslinic acid, with a significant shortcut in the number of focus with 1, 2, 3, 4 and ≥5 crypts at the dose of 25 mg/kg. MDF were markedly lower in the treated groups, with a reduction of 27% at the lower dose of 5 mg/kg that increased to 51% at 25 mg/kg. Moreover, this compound exerted a halt in the multiplicity of MDF, since the count of focus with 1, 2, 3 and 4 crypts were decreased with respect to the positive control. Taken together our results found in both, ACF and MDF, there is an unequivocal evidence of the chemopreventive potential of maslinic acid in the initiation phase of CRC. 

To the best of our knowledge, this is the first report to demonstrate the potential of maslinic acid to reduce DMH-induced ACF and MDF. This murine model of CRC has been widely used to evaluate the chemopreventive activity of other pentacyclic triterpenes such as oleanolic acid, ursolic acid and glycyrrhizic acid, among others [22,29]. In this way, oleanolic acid, that only differs from maslinic acid by the lack of a hydroxyl group at the 2-carbon position, reduced the number of ACF and crypt multiplicity [30,31]. Similar results were found for both oleanolic acid and its positional isomer, ursolic acid [32]. Moreover, glycyrrhizic acid supplementation reduced the number of ACF but not at the significant level. However, MDF were significantly reduced by the treatment [33]. One of the plausible effects of these compounds was the attenuation of the mucin depletion in DMH treated animals due to the anti-inflammatory properties of these pentacyclic triterpenes [22]. In this sense, it has been reported that maslinic acid acts as a suppressor of the pro-inflammatory pathway. This pentacyclic triterpene has been described to inhibit the transcription factor NF-κB which has been involved in the progression of inflammation linked to colon malignancies by down-regulating the expression of iNOs, and COX-2 [34,35]. These key inflammatory molecules have been reported in mucin depleted foci, thus indicating that detectable levels of local inflammation are present in the very early phases of carcinogenesis [23]. Targeting inflammation by maslinic acid provides an important strategy for cancer prevention, since abnormal expression of pro-inflammatory COX-2 have been thought to play a crucial role in colorectal cancer development [22]. Activation of the Wnt/ β-catenin pathway and the inflammation of the colonic mucosa, lead to the activation of transcription factors associated with cell proliferation such as c-Myc, c-Jun and cyclin-D1 as well as to apoptosis such as Bcl-2, Bcl-xl and p53 [22]. The effects exerted by maslinic acid on ACF and more important in the dysplastic MDF indicates a chemopreventive activity on the initiation of colon carcinogenesis. The activity developed in the early phases of carcinogenesis would reinforce those found in colorectal adenocarcinoma cell lines HT-29 and Caco-2 cells. We have previously shown that maslinic acid is capable of inhibiting the growth in HT29 cells and induced apoptosis by the intrinsic pathway, as evidenced by the generation of mitochondrial superoxide anions that served as a pro-apoptotic signal [36]. Subsequently, these results were further confirmed involving the JNK-Bid signaling pathway via the activation of p53 prior to the activation of caspases [12]. However, in Caco-2 cells the pentacyclic triterpene induced apoptosis through a death receptor-mediated apoptotic mechanism [13]. All these data suggest that maslinic acid is able to suppress the activation of multiple CRC pathways to inhibit cell proliferation and to induce apoptosis. 

Another issue upon which our study sheds light is on setting up a relationship between the dose of maslinic acid administered and the concentration of this compound reaching the colon. Hence, this knowledge could help establish appropriate doses to produce chemopreventive effects. Our results indicate that the concentrations facing the colonic mucosa 20 h after the last oral administration were around 18, 90 and 450 nmol/g for the doses of 5, 10 and 25 mg/kg, respectively. These results suggest that the doses administered can provide amounts associated with the anti-proliferative and pro-apoptotic activities exerted in HT29 and Caco-2 cells that were within the range of 10 to 250 µM [36,37]. The link between the amounts of maslinic acid at the target site and the reduction of preneoplastic lesions was assessed by means of the Pearson’s correlation coefficient. Worth mentioning the fact that maslinic acid inhibited the formation of the more advanced lesions, represented by MDF, revealing this compound as an interesting molecule for the prevention of CRC. Among the factors related to CRC risk, obesity, physical activity, inflammation and dietary habits have been suggested to play an important role. A diet with a high intake of dietary fiber, vegetables and fruits has been associated not only with a lower incidence of CRC onset [3,4] but also to improve the overall survival in patients with this disease [38]. In the present study, maslinic acid at 5 mg/kg already exerted a chemopreventive activity, and this low dose could be accomplished following a diet rich in foods containing a high amount of this compound. Thus, the intake of one serving size (125 g) of cooked lentils [39], chickpeas [39], eggplant [40], and spinaches [40] will supply 4.9, 7.7, 8.4 and 14.2 mg of maslinic acid, respectively. It is especially remarkable the high content of this triterpene in table olives, since the intake of only 7 units of the Kalamata variety will provide an amount greater than 10 mg of maslinic acid [41]. Therefore, our results not only strengthen the evidence that the intake of maslinic acid protects against CRC, but also could help to develop dietary recommendations of foods rich in this compound for the prevention of this disease. 

## 4. Materials and Methods

### 4.1. Chemicals and Reagents

Maslinic acid was provided by Dr. Parra from the University of Granada (Granada, Spain). The triterpene was obtained as a pure (>95%) white powder after extraction of the olive pomace with ethyl acetate and further purification by two-step flash chromatography [14]. Betulinic acid, which was used as internal standard (I.S.), was supplied by Extrasynthèse (Genay, France). Ethyl acetate and methanol were from J.T. Baker (Deventer, The Netherlands), whereas acetonitrile was from Scharlau Chemie S.A. (Barcelona, Spain), being all of them LC-MS grade. (2-Hydroxypropyl)-β-cyclodextrin and 10% buffered formalin (pH 7.4) were provided by Sigma-Aldrich S.L. (Tres Cantos, Madrid, Spain). All other chemicals used in the preparation of solutions were of analytical reagent grade. Ultrapure water was obtained by purification through a Milli-Q® Gradient system (Merck Milliore, Madrid, Spain).

### 4.2. Animals and Diets

Adult male rats (7–8 weeks old) of the Sprague-Dawley strain were obtained from the Animal House Facility of the Facultat de Farmàcia i Ciències de l’Alimentació (Universitat de Barcelona). Animals were housed in cages (*n* = 2‒3/cage) and maintained in a controlled environment with a dark-light cycle of 12 h, relative humidity between 40% and 70% and temperature of 22 ± 2 °C. Rats had free access to both water and food (2014 Teklad Global 14%, Harlan, Barcelona, Spain). Maslinic acid was not detected in the commercial diet analyzed following an extraction method previously described [14]. Animal manipulation was performed in the morning to avoid the effects of circadian rhythms. All experimental procedures met the ethical requirements established by the Guide for the Care and Use of Laboratory Animals and were approved by the Ethics Committee of Animal Experimentation of the Universitat de Barcelona (CEEA-UB ref. 373/12) and the Generalitat de Catalunya (ref. 6558). 

### 4.3. Experimental Design

Rats were randomly distributed into seven groups: Group DMH-/MA- (negative control: no carcinogen, no test agent; *n* = 8), Group DMH–/MA 5 (no carcinogen, 5 mg/kg of maslinic acid; *n* = 6), Group DMH−/MA 10 (no carcinogen, 10 mg/kg of maslinic acid; *n* = 6), Group DMH+/MA− (positive control: DMH, no test agent; *n* = 8), Group DMH+/MA 5 (DMH, 5 mg/kg of maslinic acid; *n* = 6), Group DMH+/MA 10 (DMH, 10 mg/kg of maslinic acid; *n* = 6) and Group DMH+/MA 25 (DMH, 25 mg/kg of maslinic acid; *n* = 6). Maslinic acid was administered daily by oral gavage (10 mL/kg) at doses of 5, 10 or 25 mg/kg over 49 days. Due to its low solubility in water, an aqueous solution of the triterpene was obtained by means of (2-hydroxypropyl)-β-cyclodextrin 20% (*w*/*v*) [10]. This solution was prepared weekly and kept at 4 °C. Rats in the groups DMH−/MA− and DMH+/MA− received only the solvent. On days 8, 15 and 22 of the experimental period, the carcinogenic agent dissolved in EDTA 1 mmol/L (pH 6.5) was administered at a dose of 20 mg/kg by intraperitoneal injections (1 mL/kg). DMH doses were freshly prepared before each use. Animals in the three DMH– groups were given an intraperitoneal injection of EDTA 1 mmol/L. 

Body weight was recorded daily, and food and water consumption were monitored every two days throughout the study. Food conversion efficiency (FCE) was calculated, as a percentage, dividing the weekly body weight gain by the weekly food consumption.

### 4.4. Sample Collection

At the end of the experimental period, overnight fasted rats were anesthetized with ketamine and xylacine (90 and 10 mg/kg, respectively), the abdomen was opened by a midline longitudinal incision and the colon of each animal was resected. The intestinal lumen was rinsed with 5 mL of ice-cold phosphate-buffered solution (PBS) to collect the intestinal content for the quantification of maslinic acid. These samples were rapidly immersed in liquid N_2_ and stored at ‒20 °C until analysis. Subsequently, colons were trimmed of mesenteric fat, cut open on the median axis and divided into three segments of similar length: proximal (close to the caecum), medial and distal (close to the rectum). The length, width and wet weight of each segment were recorded before being fixed flat onto a polystyrene board and plunged in 10% buffered formalin pH 7,4 (Sigma Aldrich, S.L.) for at least 24 h.

### 4.5. Aberrant Crypt Foci

Once fixed, colon segments were stained with histological dyes for the identification and count of preneoplastic lesions, namely aberrant crypt foci (ACF). For the evaluation of ACF, a modification of a method previously described was used [42]. Briefly, colon segments were dyed with methylene blue 0.2% for 8 min (proximal) or 10 min (medial and distal). The excess of dye was removed by rinsing the tissues with PBS and each segment was placed with the mucosal side up on a microscopic slide prior to examination at the light microscope at magnification of ×10 (Leitz, Leica Microsistemas S.L.U., Barcelona, Spain). Lesions were characterized by a larger size (2–3 times that of normal surrounding crypts), and by displaying a more intense stain, distortion of the opening of the lumen and elevation above the surface of the mucosa [42]. For each animal, the number of foci of aberrant crypts (ACF) were determined in the proximal, medial and distal segments, and were also expressed for the entire colon. Multiplicity was assessed by counting the number of aberrant crypts (AC) forming each focus (AC/ACF). Finally, the total AC in the entire colon were also calculated. The lesions were determined by two independent observers who were blinded to the treatments. 

### 4.6. Mucin Depleted Foci

Following ACF count, colon segments were kept in PBS at 4 °C until being processed with the high-iron diamine/Alcian blue/neutral red staining (HID-AB) for the observation of mucin production. Tissues were rinsed with PBS prior to being immersed in the high-iron diamine solution for 18–24 h protected from the light. Then, segments were washed in PBS prior to being stained with alcian blue 1% in acetic acid 3% for 5 min, then rinsed again, and stained for 2 min in neutral red 0.1% in acetic acid 0.002%. Tissues were mounted on microscopic slides and examined under a light microscope at magnification of ×20. The total number of mucin depleted foci (MDF), multiplicity expressed as mucin-depleted aberrant crypts per aberrant focus (MDAC/MDF) and total mucin-depleted aberrant crypts (MDAC) were assessed in the HID-AB stained colons [43]. MDF are characterized by absence or little production of mucins, distortion of the opening of the lumen as compared with normal crypts, and elevation of the lesion above the surface of the mucosa. As stated above, the scores were evaluated by two independent observers blinded to the groups.

### 4.7. Determination of AST and ALT

Aspartate aminotransferase (AST) and alanine aminotransferase (ALT) were determined in the following three representative groups: DMH−/MA− (no carcinogen, no test agent; *n* = 8), DMH+/MA− (DMH, no test agent; *n* = 8) and DMH+/MA 25 (DMH, 25 mg/kg of maslinic acid; *n* = 7). Hence, blood was collected from anesthetized rats by cardiac puncture and was transferred into a tube without anticoagulant for the determination of hepatic enzymes. Serum was obtained after centrifugation of blood samples at 1500× *g* (Megafuge 1.0R, Heraeus, Boadilla, Spain) for 15 min at 4 °C. Analyses of serum were carried out with a Roche/Hitachi 747 clinical analyzer from Roche Diagnostics GmbH (Mannheim, Germany).

### 4.8. Determination of Maslinic Acid in Colon Content

The concentration of maslinic acid in colon content was determined by liquid extraction prior to LC-APCI-MS analysis as previously described [14]. The quantification was carried out in the groups that were induced preneoplastic lesions and received maslinic acid at 0, 5, 10 and 25 mg/kg. To assess whether DMH treatment could affect the oral bioavailability of maslinic acid, the colon contents of the rats in the group that were not injected with the carcinogen but received 10 mg/kg of maslinic acid were also analyzed. In all the groups, samples were obtained between 18–20 h after the last oral administration. 

### 4.9. Statistical Analysis

Results are presented as means ± standard error of the mean (SEM). GraphPad Prism 6 (GraphPad Software, Inc., La Joya, CA, USA) was used for data evaluation and statistical analyses. Normality of the data was evaluated by the Kolmogorov-Smirnov test, and depending on the significance a parametric or non-parametric analysis was applied. Body weight and food conversion efficiency were compared using a two-way analysis of variance (ANOVA) followed by Tukey’s multiple comparisons test. The number of ACF, MDF in total colon, and segments as well as multiplicity of ACF and MDF were analyzed by the non-parametric Kruskal–Wallis test, followed by Dunn’s multiple comparisons test. AST and ALT in serum, as well as the concentration of maslinic acid in colon content were evaluated with one-way ANOVA and Bonferroni’s multiple comparisons test. The correlations between the percentage of reduction of ACF and MDF colonic content of maslinic acid were assessed by Pearson’s correlation method, previous application of Kolmogorv-Smirnov test. For all tests, two levels of significance were considered, *p* < 0.05 and *p* < 0.01.

## 5. Conclusions

The results described here demonstrate that the daily ingestion of maslinic acid at doses as low as 5 mg/kg reduces the formation of ACF and most importantly the dysplastic MDF lesions in a rat model relevant to human colorectal carcinogenesis. Consequently, and based on the results, it is not unreasonable to associate maslinic acid to the lower prevalence of CRC linked to a greater adherence to a Mediterranean diet [1,2]. Therefore, this pentacyclic triterpene merits further clinical evaluation in CRC chemoprevention.

## Figures and Tables

**Table 1:** Hepatic enzymes in animals in the negative control group, positive control group and injected with DMH and MA at 25 mg/kg treatment group ^1^.

	DMH−/MA−	DMH+/MA−	DMH+/MA 25
AST, *UI*/*L*	161 ± 20 (*n* = 8)	172 ± 30 (*n* = 8)	178 ± 15 (*n* = 6)
ALT, *UI*/*L*	54.8 ± 2.7 (*n* = 8)	53.5 ± 3.9 (*n* = 8)	54.1 ± 2.8 (*n* = 6)

^1^ Values are means ± SEM and were evaluated with one-way ANOVA and Bonferroni’s multiple comparisons test. No significant differences (*p* > 0.05) were observed between groups.
